# Dexamethasone treatment of murine auditory hair cells and cochlear explants attenuates tumor necrosis factor-α-initiated apoptotic damage

**DOI:** 10.1371/journal.pone.0291780

**Published:** 2023-09-21

**Authors:** Byung Chul Kang, Junyeong Yi, Song Hee Kim, Jhang Ho Pak, Jong Woo Chung

**Affiliations:** 1 Department of Otorhinolaryngology-Head and Neck Surgery, Ulsan University Hospital, University of Ulsan College of Medicine, Ulsan, Korea; 2 Department of Otorhinolaryngology-Head and Neck Surgery, Asan Medical Center, University of Ulsan College of Medicine, Seoul, Korea; 3 Department of Convergence Medicine, University of Ulsan College of Medicine and Asan Institute for Life Sciences, Asan Medical Center, Seoul, Korea; Universidade Federal de Sao Paulo/Escola Paulista de Medicina (Unifesp/epm), BRAZIL

## Abstract

The most common cause of sensorineural hearing loss is damage of auditory hair cells. Tumor necrosis factor-alpha (TNF-α) is closely associated with sensorineural hearing loss. The present study examined the preconditioning effect of dexamethasone (DEX) on TNF-α-induced ototoxicity in mouse auditory hair cells (HEI-OC1) and cochlear explants. Treatment of HEI-OC1 with 10 ng/ml TNF-α for 24 h decreased cell viability, increased the accumulation of reactive oxygen species (ROS), and induced caspase-mediated apoptotic signaling pathways. Pretreatment with 10 nM DEX for 6 h before TNF-α exposure restored cell viability, decreased ROS accumulation, and attenuated apoptotic signaling activation induced by TNF-α. Incubation of cochlear explants with 20 ng/ml TNF-α for 24 h resulted in significant loss of both inner hair cells (IHCs) and outer hair cells (OHCs) and an increase in apoptotic activation accessed by annexin V staining. The cochlear explants pre-incubated with 10 nM DEX attenuated TNF-α ototoxicity in both IHCs and OHCs and apoptotic cell death. These results indicated that DEX plays a protective role in ototoxicity induced by TNF-α through attenuation of caspase-dependent apoptosis signaling pathway and ROS accumulation.

## Introduction

The most common cause of sensorineural hearing loss is damage of auditory hair cells. Many causes including aging process, noises, ototoxic drugs, trauma, vascular disorders, viral infections, and immune-related mechanisms act as insults to the auditory hair cell and result in sensorineural hearing loss [1–3]. Because the exact cause of sudden sensorineural hearing loss is unknown, and given several potential etiologies, various factors that affect prognosis and treatment have also been proposed [4–6]. Changes in the serum levels of cytokines, such as tumor necrosis factor-α (TNF-α) and interleukin-6 (IL-6), have been related with poor prognosis in patients with sudden sensorineural hearing loss [7]. Reports show that posttreatment serum TNF-α levels are higher than pretreatment levels in patients non-responsive to the treatment of sudden sensorineural hearing loss [8]. Although the precise molecular mechanism remains to be elucidated, studies suggest that the serum level of TNF-α may play a key role in the pathophysiology of sudden sensorineural hearing loss. Whether the elevated level of TNF-α is the cause of or the consequence of sensorineural hearing loss, TNF-α blockade is expected as an avenue for treatment of immune-mediated hearing loss.

TNF-α is a well-established proinflammatory cytokine that can induce apoptosis in various types of cells [9, 10]. TNF-α activates both extrinsic cell death and intrinsic signaling pathways; the former is mediated by the TNF receptor-1 (TNFR1) [11], and the latter involves alterations in the expression of an anti-apoptotic protein (B-cell lymphoma 2, Bcl-2) and a pro-apoptotic protein group (Bcl-2-associated X family, Bax) [12]. These pathways work together and subsequently activate procaspases for inducing cell death. TNF-α has been identified as a crucial cytokine in the inner ear [13]. Several animal and clinical studies have reported an association between increased levels of TNF-α with hearing loss and the protective effects of TNF-α inhibitors on hearing loss [14–16], although it is still controversial whether TNF-α acts as a mediator or is a direct etiology of hearing loss.

Corticosteroids or synthetic glucocorticoids have been used to treat various inner ear diseases, including sudden sensorineural hearing loss, immune-related hearing loss, Ménière’s disease, and inner ear diseases [17–19]. Intratympanic steroid injection is used as initial or salvage therapy for inner ear diseases because sufficient concentration is delivered to the inner ear through a round window [18, 20]. Corticosteroids can defend the inner ear against cytokines such as TNF-α and IL-1β by inhibition of IL-1β-induced matrix metalloproteinase (MMP)-9 expression, activation of NF-κB and upregulation of TNFR1 [21, 22]. Therefore, the relationship between TNF-α and corticosteroid needs to be evaluated.

The present study examined the effects of TNF-α on cell viability, accumulation of reactive oxygen species (ROS), and changes in apoptosis-related protein expression in mouse auditory hair cells (HEI-OC1). Moreover, we investigated whether the beneficial effects of dexamethasone (DEX) treatment on TNF-α could apply to mouse cochlear explants.

## Materials and methods

### Materials

The components of the cell culture medium were procured from Thermo Fisher Scientific (Waltham, MA, USA) unless indicated otherwise. TNF-α and DEX were bought from BioLegend (575204, San Diego, CA, USA) and Enzo Life Sciences (BML-EI126, Farmingdale, NY, USA), respectively. Polyclonal antibodies against Bcl-2 (3498), caspase-3 (9662), cleaved caspase-3 (9661), cleaved caspase-7 (9491), and poly (ADP-ribose) polymerase (PARP)/cleaved PARP (9542) were purchased from Cell Signaling Technology (Danvers, MA, USA), Bax (556467) from BD Biosciences (Franklin Lakes, NJ, USA), caspase-7 (sc-81654) from Santa Cruz Biotechnology (Santa Cruz, CA, USA), and β-actin (A5441) from Sigma-Aldrich (St. Louis, MO, USA). Horseradish peroxidase (HRP)-conjugated secondary antibodies were purchased from Bethyl Laboratories (Montgomery, TX, USA).

### Cell culture and TNF-α/DEX treatment

The immortalized HEI-OC1 mouse auditory cells were kindly provided by Dr. Federico Kalinec (Department of Cell and Molecular Biology, House Ear Institute, Los Angeles, CA, USA). The HEI-OC1 cells were previously characterized in a publication [23]. The HEI-OC1 cells were maintained in high glucose Dulbecco’s modified Eagle’s medium (DMEM) supplemented with 10% fetal bovine serum (FBS) in a humidified chamber containing 10% CO_2_ at 33°C. For experiments involving TNF-α or/and DEX treatment, cells were seeded into appropriate culture dishes and incubated for 24 hours under standard conditions. After 3 hours incubation in a serum-free medium, cells were treated to various concentrations of TNF-α or DEX. In DEX pretreatment studies, cells were pretreated with 10 nM DEX for 6 hours. DEX containing culture medium was discarded, and cells were treated with 10 ng/ml TNF-α for additional 24 hours.

### Cell viability assay

A colorimetric D-Plus CCK cell viability assay kit (Dongin LS, Seoul, South Korea) was utilized to evaluate the viability of cells following to the manufacturer’s protocol. Cells were seeded in 96-well plates at a density of 4 × 10^3^ cells/well and incubated under standard conditions for 24 hours. The serum-starved cells were treated to various concentrations of TNF-α (0–20 ng/ml), DEX (0–20 nM), or DEX (10 nM) plus TNF-α (10 ng/ml). The amount of formazan dye product was established by measuring absorbance at 450 nm with a microplate spectrophotometer (Molecular Devices, San Jose, CA, USA). The absorbance values were converted into percentages relative to the untreated control.

### Measurement of intracellular ROS

The quantification of intracellular ROS was conducted using 5-(and-6)-chloromethyl-2′-7′-dichlorodihydrofluorescein diacetate, acetyl ester (CM-H2DCFDA; Invitrogen, Waltham, MA, USA). Cells were seeded in 96-well plates and then incubated with DEX, TNF-α, or both sequentially, as described previously. Subsequently, cells were washed with Hank’s balanced salt solution (HBSS) and incubated with 5 μM CM-H2DCFDA for 20 minutes at 33°C in the dark. For quantification of ROS levels, DCF fluorescence was determined by a spectrofluorometer (VICTOR 3 fluorescence reader, Perkin-Elmer, Waltham, MA, USA) at 485 nm (excitation) / 535 nm (emission).

### Immunoblotting

Total protein was extracted by supplementing the RIPA lysis buffer (Sigma-Aldrich) with protease and phosphatase inhibitors. Protein concentrations were quantified with the Pierce BCA Protein Assay Kit (Thermo fisher). After separating equal amounts of protein via sodium dodecyl sulfate-polyacrylamide gel electrophoresis (SDS-PAGE), they were transferred to nitrocellulose membranes (GE Healthcare, Chicago, IL, USA). Next, membranes were washed thrice with Tween 20-containing TBS (TBST), blocked with 5% skim milk, and incubated overnight with the appropriate primary antibodies. After three washes with TBST, the membranes were incubated for 1 hour with HRP-conjugated secondary antibodies. The immunoreactive bands were developed by an enhanced chemiluminescence assay kit (ECL; Dongin LS) and detected by the ImageQuant LAS 500 biomolecular imager (GE Healthcare).

### Detection of apoptosis by Annexin V/propidium iodide (PI) staining

Annexin V/PI staining was carried out in accordance with the manufactor’s instruction using an Annexin V-FITC Apoptosis staining/Detection kit (Abcam, Cambridge, UK). The cells cultured on coverslips in 6-well plates were treated with TNF-α or 10 nM DEX for 6 hours followed by TNF-α, and further incubated for 24 hours. After washing with binding buffer, cells were incubated for 10 minutes with Annexin V-FITC and PI solution in the dark. Cells were mounted on glass slides and visualized using the appropriate filter of an Olympus IX71 fluorescence microscope. Green fluorescence (ex/em ∼495/515 nm) was used to detect early apoptotic cells, while red fluorescence (ex/em ∼561/615 nm) was used to detect late apoptotic and necrotic cells.

### Animal experiments

The protocol of animal experiments was approved by the Committee on Use and Care of Animals of the University of Ulsan (IACUC No. 2021-12-268; approval date, October 05, 2021). The animal experiments were performed according to the guidelines of the National Institutes of Health and the principles of the Declaration of Helsinki. All the animal care was conducted under the supervision of the Laboratory Animal Unit of the Asan Institute for Life Sciences (Seoul, Korea). The pregnant C57BL/6J mice (9–13 weeks old, 20–25 g) were obtained from Central Laboratory Animal Inc. (Seoul, Korea), and 3-days neonatal mice were used for explant culture studies. All the neonatal mice were euthanized by decapitation to collect their cochleae, and all efforts were made to minimize suffering.

### Cochlear explant cultures

To provoke the auditory hair cell damage under more physiologic condition, murine cochleae were isolated. Mice of postnatal day 3 (P3) were euthanized via decapitation, and ∼ 40 cochleae were carefully dissected in ice-cold HBSS solution. The stria vascularis, spiral ligament, and the Reissner’s membrane were meticulously eliminated to isolate cochlear explants. The middle-turn cochlear explants were plated onto 4-well culture dishes and incubated in culture media (98% DMEM, 1% N-2 supplement, and 1% ampicillin) at 37°C in 5% CO_2_ overnight. Culture medium was substituted with fresh medium (control) or medium containing different concentrations of TNF-a (10–40 ng/ml) and incubated for 24 hours. For DEX-mediated inhibitory experiment, explants were incubated with medium containing 10 nM DEX for 6 hours prior to treatment with 20 ng/ml TNF-a.

### Immunofluorescence staining

After washing twice with PBS and the cochlear explants fixed with 4% paraformaldehyde for 20 minutes. The explants were permeabilized in 0.5% Triton X-100, blocked in 5% normal goat serum (NGS), and incubated overnight with the primary antibody against myosin 7a (1:100, sc-74516, Santacruz) at 4°C. Explants were then washed three times with NGS and incubated for 1.5 hours with Alexa Fluor 564 goat anti-mouse secondary antibody (1:200, A11005, Invitrogen). After washing with NGS and distilled water, explants were counterstained with DAPI for 5 minutes and briefly washed with distilled water. Finally, explants were dehydrated in the air and mounted with fluorescence mounting medium (S3023, Agilent Technologies, CA, USA). Images of the stained middle-turn cochlear explants were observed with a Zeiss LSM 880 confocal microscope (Carl Zeiss, Oberkochen, Germany).

For Annexin V staining, permeabilized explants were washed with PBS and binding buffer and were incubated for 10 minutes with buffer containing Annexin V-FITC in the dark. The procedure of myosin 7a staining and the observation of fluorescent images were performed as described above. The numbers of myosin 7a and annexin V positive inner hair cells (IHCs) and outer hair cells (OHCs) were counted over a distance of 160 um in the middle turn of each cochlear explant.

### Statistical analysis

The data are presented as means ± standard error (SE) from three or four independent experiments. Statistical analyses were carried out by Student’s t-test or one-way analysis of variance (ANOVA) using SigmaPlot software (Ver.12.0; Jandel Scientific, San Rafael, CA, USA). A p-value less than 0.05 was considered statistically significant.

## Results

### Effects of TNF-α and DEX on cell viability

**Fig 1:**
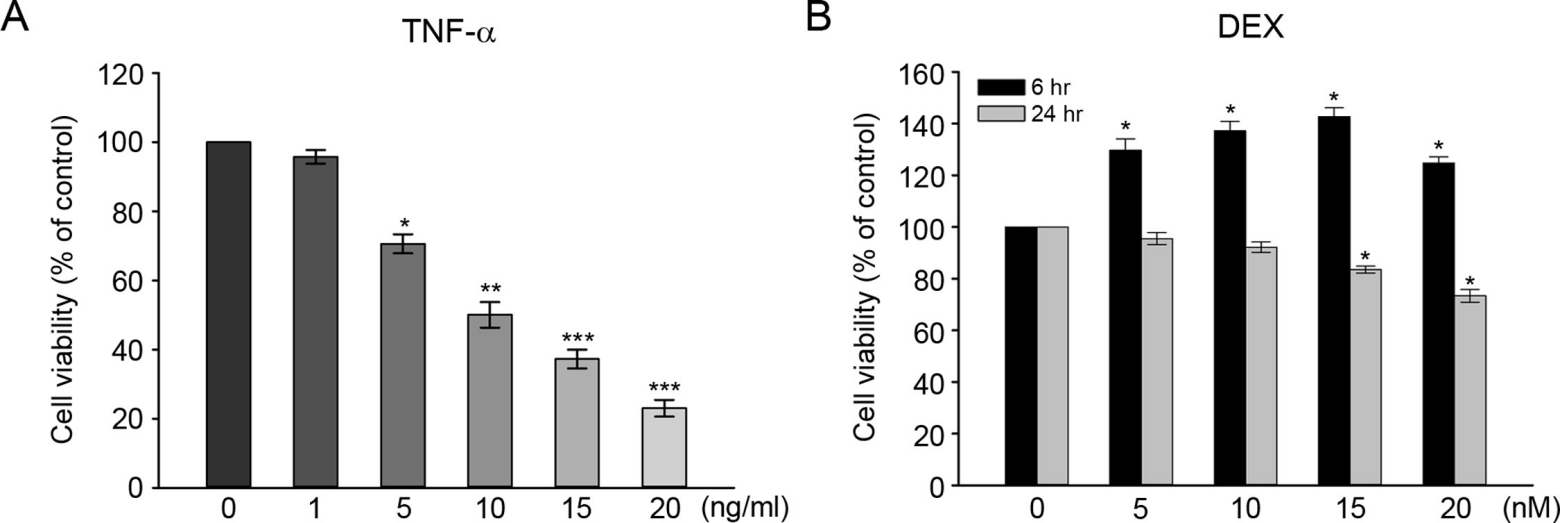
Effects of TNF-α and dexamethasone (DEX) on cell viability (A) Dose-dependent cell viability of HEI-OC1 cells exposed to TNF-α. Cells were treated with various concentrations of TNF-α (0–20 ng/ml) for 24 h and viability was measured using a CCK-8 assay. (B) Cell viability of HEI-OC1 cells at various concentrations of DEX. Cells were treated with DEX (0–20 nM) for 6 h or 24 h and viability was measured using a CCK-8 assay. All values are expressed as the means ± SE for four independent experiments, expressed as a percentage of untreated control value. * P < 0.05, ** P < 0.01, *** P < 0.001 compared with untreated control.

Reports show that various cells exposed to TNF-α induce apoptotic cell death [24, 25]. To determine the cytotoxic effect of TNF-α on auditory hair cells, HEI-OC1 cells were treated with different concentrations of TNF-α (1, 5, 10, 15, 20 ng/ml) for 24 hours. As shown in Fig 1A, TNF-α treatment dose-dependently decreased cell viability. At 10 ng/ml TNF-α concentration, cell viability was ∼50% (estimated half-maximal cytotoxic dose) compared to untreated control. Thus, we used 10 ng/ml TNF-α concentration in subsequent experiments. To determine the effect of DEX on auditory hair cells, HEI-OC1 cells were also treated with different concentrations of DEX (5, 10, 15, and 20 nM) for 6 and 24 hours. At 6 hours after DEX treatment, cell proliferation was increased at all doses and reached a maximum peak at 15 nM (Fig 1B). However, the 24-hours treatment produced decreased cell viability at 15 and 20 nM concentrations. This result indicated that treatment with 10 nM of DEX for 6 hours was adequate for DEX-mediated protection experiments against TNF-α.

### Effects of TNF-α and DEX on ROS generation and expression of apoptosis-related proteins

**Fig 2:**
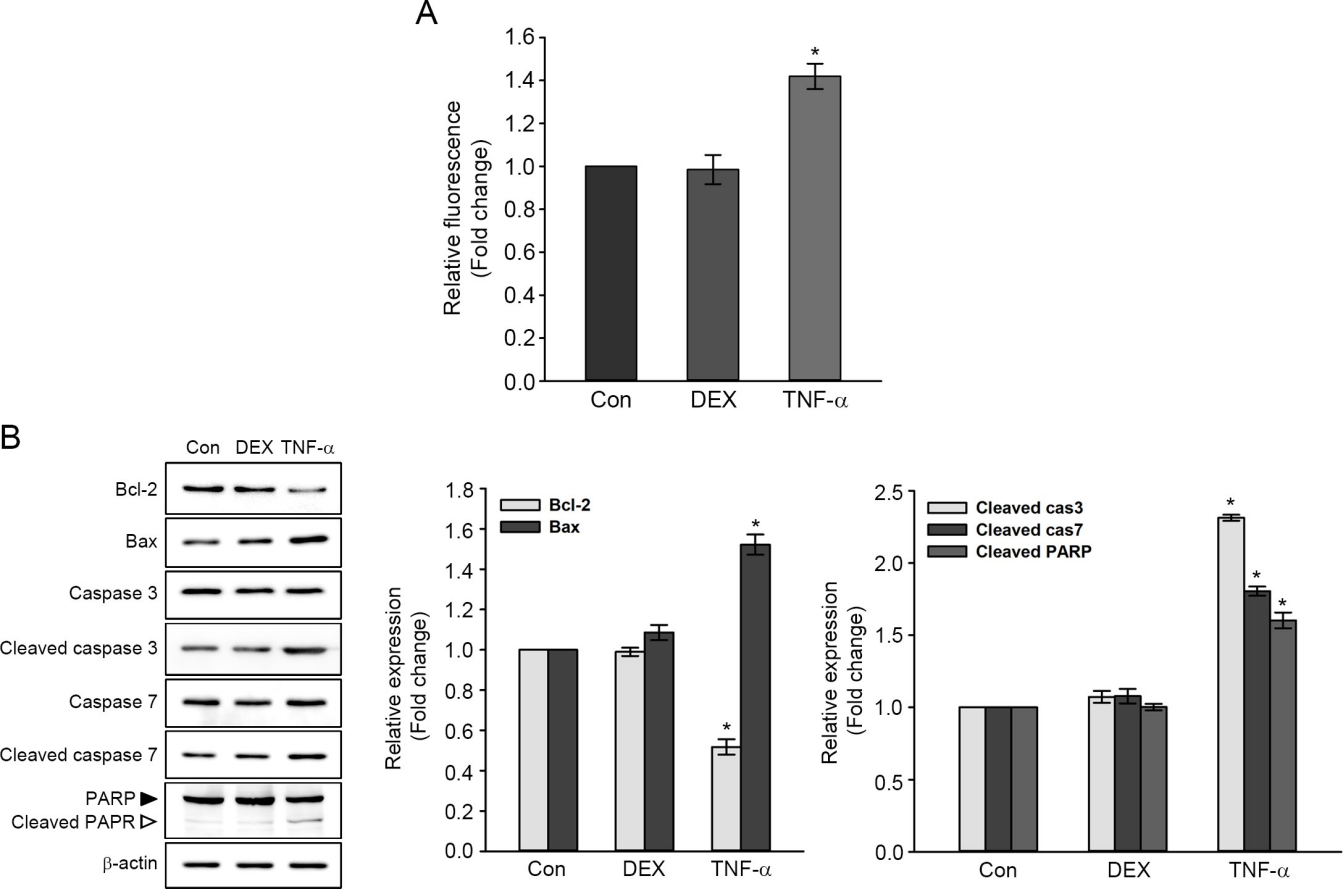
Effects of DEX and TNF-α on intracellular ROS accumulation and expression of apoptosis-related proteins. Cells were treated with 10 nM DEX for 6 h or 10 ng/ml TNF-α for 24 h. (A) Measurement of ROS generation using DCF fluorescence intensities. Values are expressed as the means ± SE for three independent experiments, expressed as a fold change of untreated control value. * *P* < 0.05 compared with the untreated control. (B) Representatives immunoblot of apoptosis-related protein expression. Individual band was quantified by densitometry and normalized to β-actin expression. The values in graphs are represented as fold changes relative to the untreated control, expressed as means ± SE of three independent experiments. * *P* < 0.05 compared with untreated control.

Fig 2A shows the changes in intracellular ROS levels measured by DCF fluorescent probe. Intracellular accumulation of ROS was significantly higher by ∼1.40-fold in TNF-α-treated cells than in untreated control (Fig 2A). However, there was no definite change in ROS levels between DEX-treated cells and untreated control.

The expression of apoptosis-related proteins is shown in Fig 2B. The levels of the anti-apoptotic protein Bcl-2 and the pro-apoptotic protein Bax in DEX-treated cells were not significantly different from those of untreated control. The levels of cleaved caspase-3 and -7 (catalytically active forms of caspases) and cleaved PARP fragments (an inactivated form of PARP) were also not significantly changed by DEX treatment. With the treatment of TNF-α, the level of Bcl-2 decreased by ∼0.52-fold, and the level of Bax increased by ∼1.52-fold compared to the untreated control. The cleaved caspase-3, -7, and PARP levels increased in TNF-α-treated cells by ∼2.31-, ∼1.81-, and ∼1.60-fold, respectively, compared to untreated control. These results showed that intracellular ROS accumulation and caspase-mediated apoptotic signaling pathways were induced by TNF-α in HEI-OC1 cells but these were not affected by DEX.

### Protective effects of DEX on TNF-α-induced auditory hair cell damage

**Fig 3:**
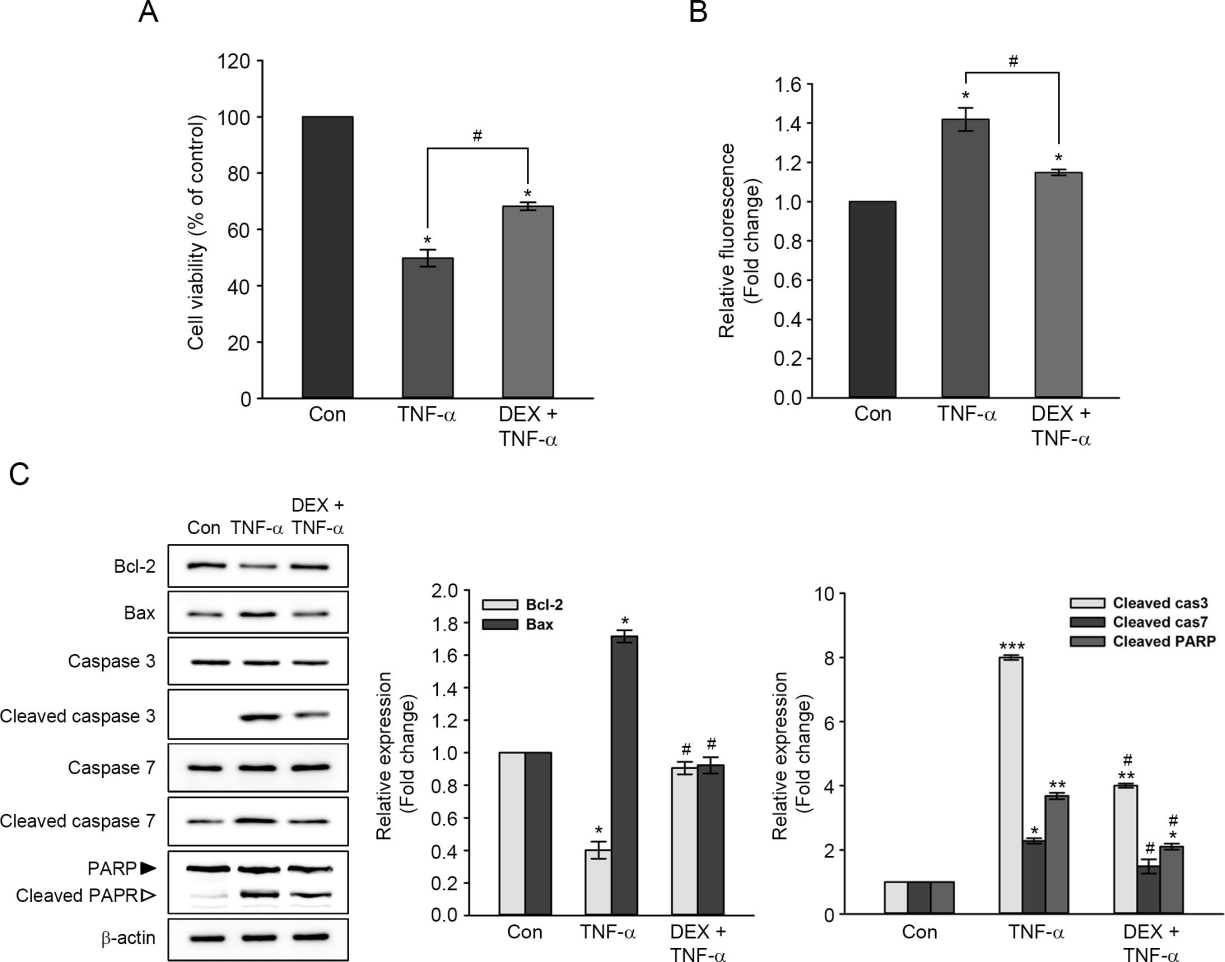
Protective effect of DEX on cell viability, ROS generation, and expression of apoptosis-related proteins in TNF-α-treated cells. Cells were pretreated with 10 nM DEX for 6 h, followed by treatment with 10 ng/ml TNF-α for a further 24 h. (A) The effect of DEX pretreatment on cell viability reduced by TNF-α. Data in the graph are expressed as means ± SE of four independent experiments. *^,#^ *P* < 0.05 *; compared with untreated control, ^#^; TNF-α only versus DEX plus TNF-α. (B) Measurement of intracellular ROS accumulation using CM-H_2_DCFDA. Values are expressed as means ± SE for three independent experiments. *^,#^ *P* < 0.05 *; compared with untreated control, ^#^; TNF-α only versus DEX plus TNF-α. (C) Representatives immunoblot of apoptosis-related protein expression. Each protein expression was normalized by that of β-actin. The values in a graph are expressed as fold changes relative to the untreated control and expressed as means ± SE of three independent experiments. *^,#^ *P* < 0.05, ** *P* < 0.01, *** *P* < 0.001, *; compared with untreated control, ^#^; TNF-α only versus DEX plus TNF-α.

To investigate the protective effect of DEX on TNF-α-induced cytotoxicity, HEI-OC1 cells were pretreated with 10 nM DEX for 6 hours, followed by treatment with 10 ng/ml TNF-α for a further 24 hours. DEX pretreatment restored cell viability by ∼20%, which was reduced by TNF-α-treatment (Fig 3A). Concomitantly, intracellular ROS levels induced by TNF-α were significantly decreased in DEX-pretreated cells (Fig 3B). Fig 3C shows the apoptosis-related protein expression. The level of Bcl-2 expression was reduced by ∼0.40-fold in TNF-α-treated cells. DEX pretreatment reduced TNF-α-induced Bcl-2 expression reduction, showing levels similar to those of untreated controls. The level of Bax expression increased by ∼1.72-fold in TNF-α-treated cells. In the cells pretreated with DEX, TNF-α-induced increase of Bax expression was decreased to untreated control level. Also, the activation of caspase-7 induced by TNF-α significantly attenuated to untreated control level by DEX pretreatment. The activation of caspase-3 and inactivation of PARP induced by TNF-α were also significantly attenuated by DEX pretreatment, but these were still higher than the control level (Fig 3C).

**Fig 4:**
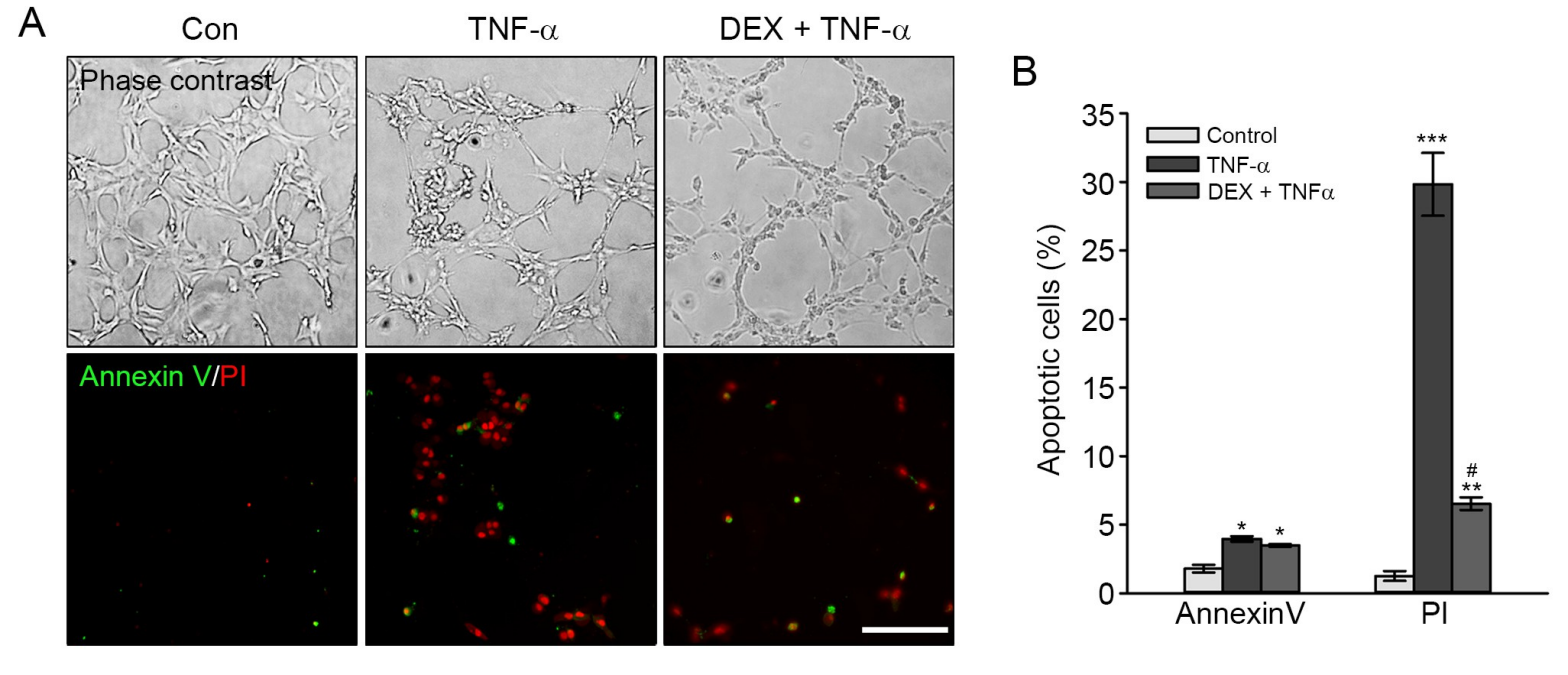
Attenuation of TNF-α-induced apoptosis by DEX pretreatment. Cells were pretreated with DEX for 6 h before exposure to TNF-α for 24 h. (A) Representative images of Annexin V (green) and PI (red) staining in HEI-OC1 cells. Scale bar = 100 μm, Original magnification = 100 ×. (B) Values in the graphs are presented the percentage of apoptotic cells. The data in graphs are expressed as means ± SE (*n* = 3). *^,#^ *P* < 0.05, ** *P* < 0.01, *** *P* < 0.001, *^,^**^,^***; compared with untreated control, ^#^; TNF-α only versus DEX plus TNF-α.

Finally, to determine the inhibitory effect of DEX on TNF-α-induced apoptosis, we performed the Annexin V/PI staining assay. As shown in Fig 4, the percentages of early apoptotic cells (green) observed by the fluorescence microscope were ∼3.97% in cells treated with TNF-α and ∼3.50% in DEX-pretreated and TNF-α-treated cells. Also, the percentage of late apoptotic/necrotic cells (red) stained with PI was ∼29.82% in TNF-α-treated cells, whereas DEX-pretreated and TNF-α-treated cells showed the percentage of late/necrotic cells (6.53%). These results indicated that DEX pretreatment attenuated TNF-α-induced cell death in HEI-OC1 cells.

### TNF-α-induced auditory hair cell damage in cochlear explants

**Fig 5:**
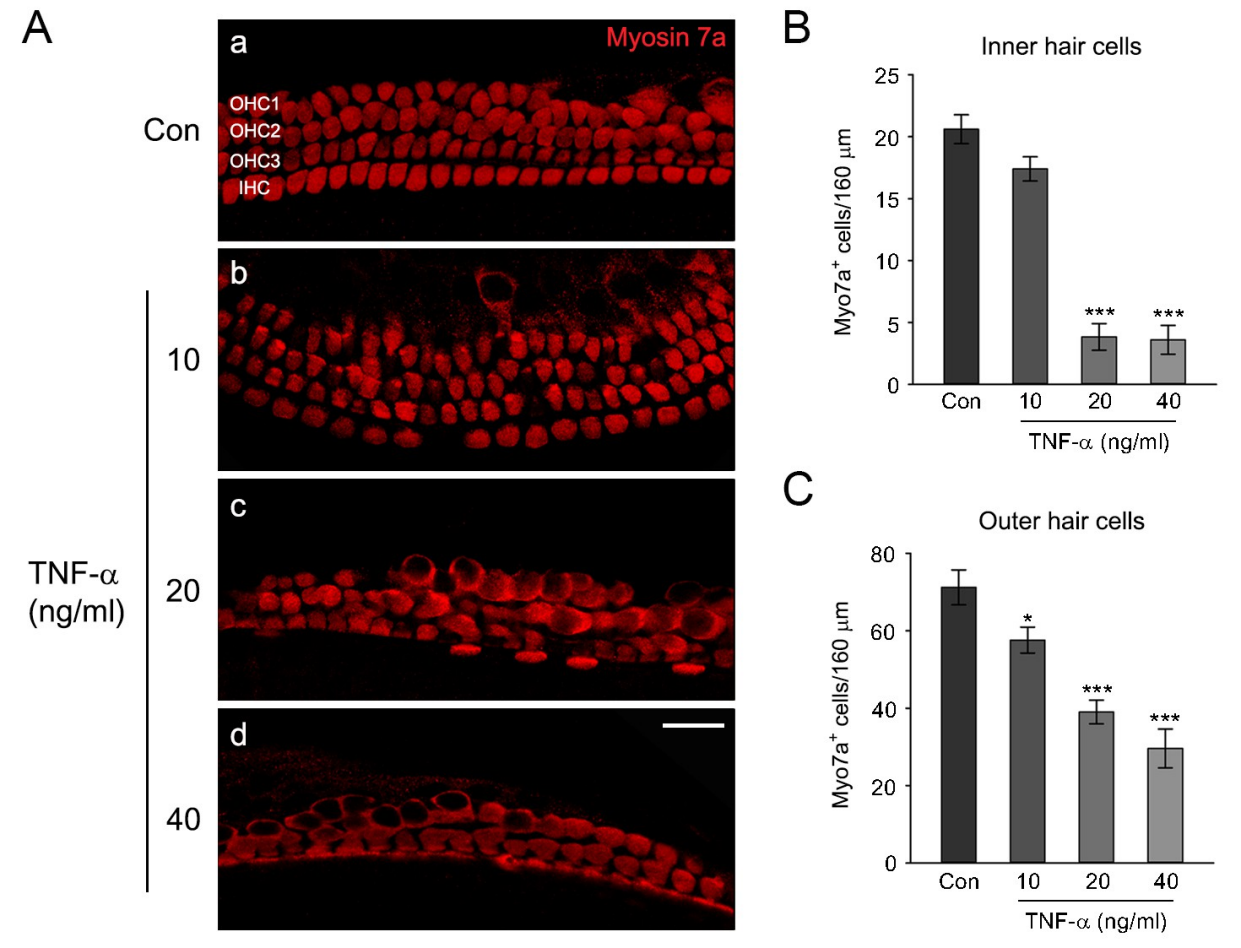
Effects of TNF-α on the cochlear hair cells in middle-turn cochlear explants. (A) The representative confocal images show the middle-turn cochlear explants treated with a culture medium alone (control) or a medium containing different concentrations of TNF-α (10, 20, and 40 ng/ml). After treatment, explants were fixed, permeabilized, and stained with a polyclonal myosin 7a antibody as a hair cell marker. Scale bars = 20 mm, Original magnification = 400 ×. (B, C) Quantification of myosin 7a positive Inner hair cells (IHCs) and Outer hair cells (OHCs) per 160 mm in the middle-turn cochlear explants, respectively. Data are expressed as mean ± SE of the number of IHCs or OHCs (*n* = 3 different explants per group); * *P* < 0.05, *** *P* < 0.001, *^,^***; compared with untreated control.

Fig 5A shows the isolated murine cochlear explant. Normal hair cell morphology of a cochlear explant was intact, with three layers of OHCs and a single row of IHCs arranged in organized arrays (Fig 5A). Exposure to TNF-α resulted in the disruption of hair cell integrity dose-dependently. The layers of OHCs were impaired at 10 ng/ml TNF-α and disarrayed, with many OHCs lost at higher concentrations. Severe disruption of a single IHC row was evident at 20 ng/ml or higher doses (Fig 5B and 5C). Since treatment with TNF-α (20 ng/ml) significantly damaged both IHCs and OHCs of cochlear explants, we used this concentration in our subsequent experiments.

### Otoprotective effects of DEX on TNF-α-induced auditory hair cell damage in cochlear explants

**Fig 6:**
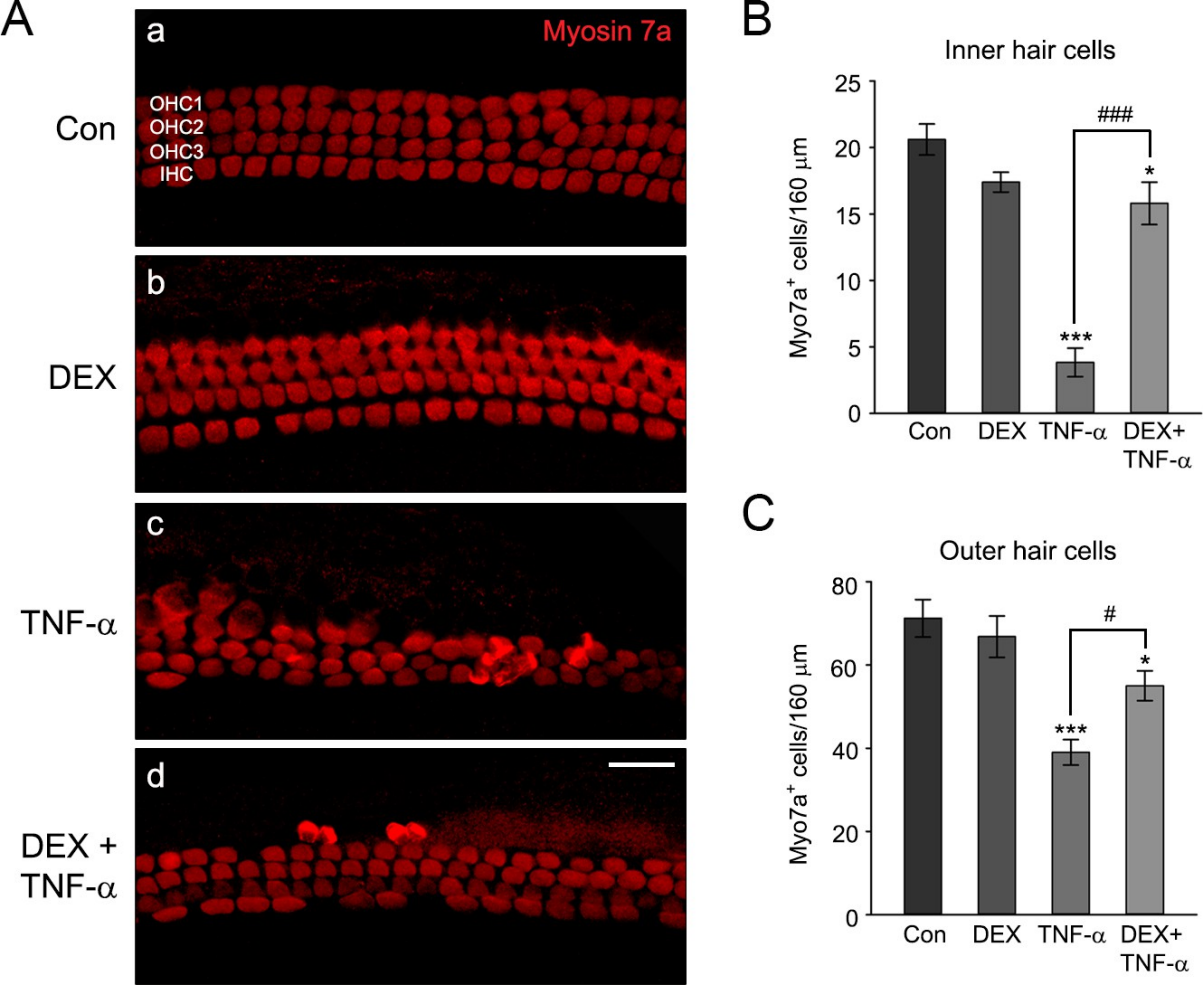
Protective effects of DEX on TNF-α-induced cochlear hair cells loss in cochlear explants. (A) The Representative confocal images of IHCs and OHCs stained by myosin 7a in middle-turn cochlear explants (a; control, b; 10 nM DEX, c; 20 ng/ml TNF-α, d; 10 nM DEX pretreatment then 20 ng/ml TNF-α). Scale bars = 20 mm, Original magnification = 400 ×. (B,C) The graph represents the number of myosin 7a positive IHCs or OHCs per 160 mm in the middle-turn cochlear explants, expressed as a mean ± SE (*n* = 3 different explants per group); *^,#^ *P* < 0.05, ***^,###^ *P* < 0.001, *^,^***; compared with untreated control, ^#,###^; TNF-α only versus DEX plus TNF-α.

**Fig 7:**
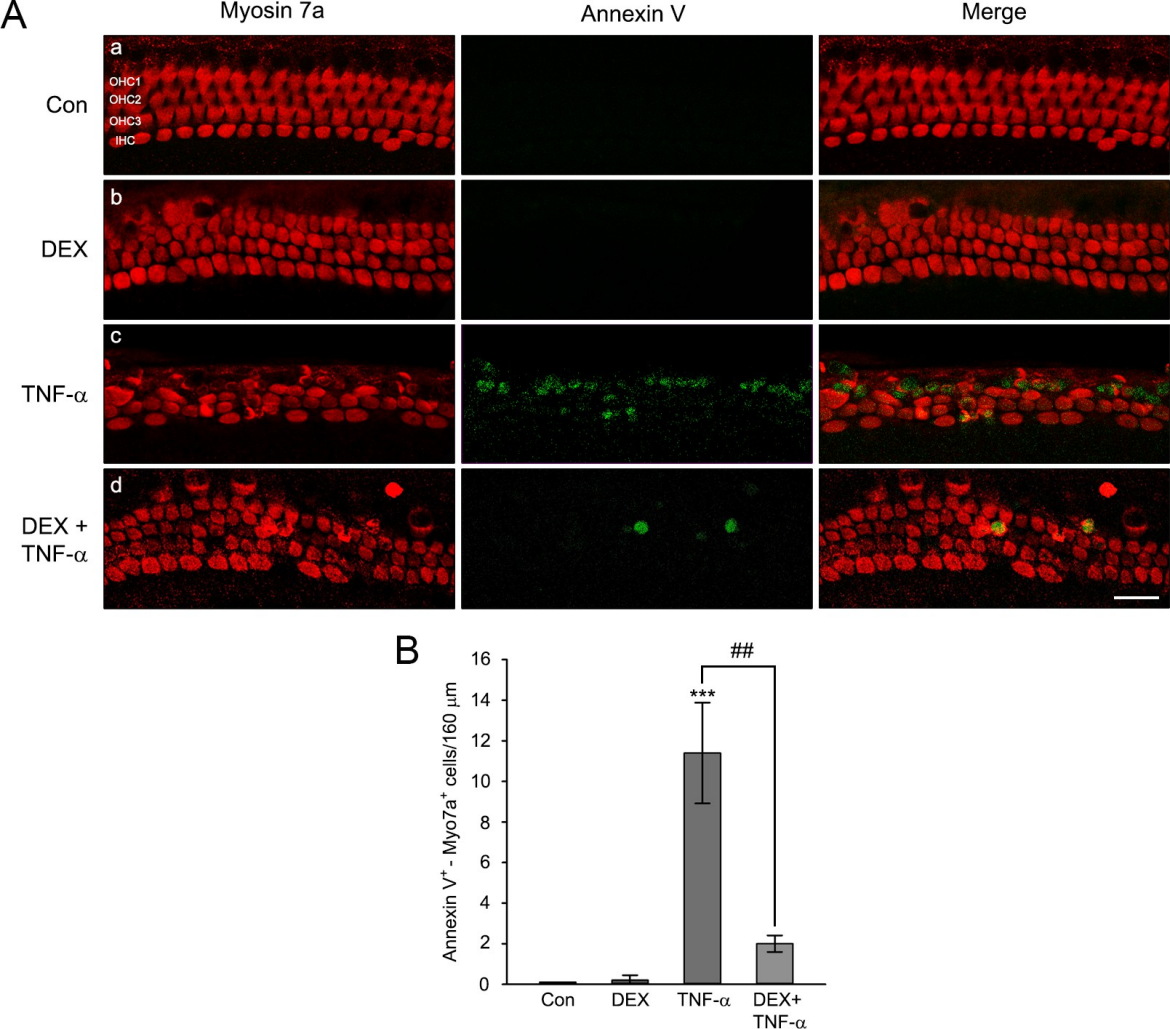
Attenuation of TNF-α-induced apoptosis through pretreatment of DEX in cochlear explants. (A) The representative confocal images showing IHCs and OHCs stained with primary antibody to myosin 7a and annexin V in middle-turn cochlear explants (a; control, b; 10 nM DEX, c; 20 ng/ml TNF-α, d; 10 nM DEX pretreatment then 20 ng/ml TNF-α). The Myosin 7a (red) and annexin V (green) were used for counting the number of hair and apoptotic cells, respectively. Scale bars = 20 mm, Original magnification = 400 ×. (B) The graph represents the number of annexin V/myosin 7a double-positive HCs per 160 mm in the middle-turn cochlear explants. Data expressed as mean ± SE of the number of HCs (*n* = 4 different explants per group); ^##^ *P* < 0.01, *** *P* < 0.001, ***; compared with untreated control, ^##^; TNF-α only versus DEX plus TNF-α.

The otoprotective effect of DEX against TNF-α-induced hair cell loss is shown in Fig 6. There were no definite morphological changes between untreated control and the explants exposed to 10 nM of DEX for 6 hours. The hair cell integrities were well-maintained in both groups. Treatment of the explants with 20 ng/ml TNF-α for 24 hours resulted in significant disarray, with the loss of many OHCs and IHCs of cochlear explants, while DEX pretreatment (for 6 hours before exposure to TNF-α) markedly reduced the degree of damage (Fig 6A). Compared with TNF-α-treated explants, the number of IHCs and OHCs increased by DEX pretreatment (Fig 6B and 6C). Fig 7 shows the staining results with annexin V and primary myosin 7a antibody. The number of apoptotic cells was dramatically increased in TNF-α-treated cochlear explants. However, DEX pretreatment significantly restored the apoptotic hair cell death and the morphological disruption (Fig 7). These results indicated that DEX pretreatment protected cochlear hair cells from auditory cell death induced by TNF-α in *ex vivo*.

## Discussion

Immune-related hearing loss is characterized by frequent recurrence and a chronic course and mediated by autoimmune response [19]. Reports show that some cytokines mediate immune responses in the pathophysiology of immune-related hearing loss. For example, elevated levels of cytokines, including IL-1, IL-17, interferon-gamma (IFN-γ), and TNF-α, have been detected in immune-related hearing loss [19, 26, 27]. Studies in animals have demonstrated a correlation between increased TNF-α level and hearing loss [13, 15, 28]. These reports indicate that TNF-α specifically plays a key role in damage of auditory hair cells, among other proinflammatory cytokines, assuming that overexpressed TNF-α is associated with hearing loss. Therefore, the blockade of TNF-α could be an important strategy in prevention and treatment of hearing loss. The present study showed that TNF-α treatment reduces cell viability and promotes accumulation of intracellular ROS in auditory hair cells, leading to caspase-dependent apoptosis. Also, DEX pretreatment attenuated TNF-α-induced damage in HEI-OC1 cells and cochlear explants.

TNF-α-mediated signaling involves two distinct pathways: the prosurvival/proinflammatory pathway and the pro-apoptotic pathway. The former activates NF-κB and MAPK through TNF-α-induced complex I. The latter stimulates production of ROS and the caspase cascade activation in mitochondria through TNF-α-induced complex II [10]. This study found that TNF-α treated-HEI-OC1 cells resulted in decreased Bcl-2 expression, increased Bax expression, and activation of caspase-3 and -7, and fragmented PARP (Fig 2). These results showed that treatment of TNF-α activated apoptotic signal pathways in auditory hair cells. Reports showed TNF-α-mediated damage in auditory hair cells and cochlea [29, 30], and a correlation between hearing loss and elevated TNF-α level in patients with immune-mediated sensorineural hearing loss [31]. Moreover, TNF-α treatment resulted in the stimulation of ROS generation in HEI-OC1 cells (Fig 2A). This result was consistent with a previous report, in which ROS modulator and Bcl-X_L_ were associated with production of ROS in reaction to TNF-α [10]. Therefore, it is reasonable to postulate that treatment of auditory hair cells with TNF-α triggers excessive ROS production through disruption of mitochondrial membrane potential, subsequently contributing to apoptotic cell death.

DEX, a synthetic corticosteroid agonist, functions as an inflammation and immune responses regulator. In general, glucocorticoid induction is mediated by a glucocorticoid receptor that binds to the glucocorticoid, then translocates to the nucleus, and binds to a glucocorticoid response element within the promoter regions [32]. Reports show that exposure to relatively high concentrations of DEX (i.e., over 1 μM) decreased cell viability in different cell lines, accompanied by the accumulation of ROS and apoptosis [33, 34]. Similar to previous studies, we found decreased HEI-OC1 cell viability treated with DEX (over 15 nM) for 24 hours. However, 6 hours treatment of 10 nM DEX in HEI-OC1 cells resulted in increased proliferation, in which apoptotic cell death and ROS accumulation were abolished (Figs 1B and 2). Consistent with this result, the human fetal osteoblastic cells and corneal epithelial cells exposed with low DEX concentrations (0.1 and 10 nM) for 24 hours can increase cell proliferation [24, 25]. Thus, our data and other reports suggest that cell proliferation or toxicity mediated by DEX depends on cell types and their concentrations.

Previous reports have shown an anti-apoptotic effect of DEX in various types of cells. For example, DEX pretreatment decreased NADPH oxidase-mediated generation of ROS in BV-2 microglial cells treated to lipopolysaccharides [35] and suppressed tunicamycin-induced apoptosis via inhibiting the expression of endoplasmic reticulum stress-related proteins in HEI-OC1 cells [36]. Also, primary cultured hepatocytes pretreated with DEX prevented TNF-α plus actinomycin D-induced caspase-dependent apoptosis [37]. Co-treatment with TNF-α and DEX effectively blocked TNF-α-induced apoptosis by inhibiting XIAP, c-IAP1, and c-IAP2 cleavage in human breast cancer cells (MCF-7) [9]. The present study indicated that DEX pretreatment attenuated TNF-α-induced cytotoxicity, probably resulting from the inhibition of excessive accumulation of ROS and apoptotic signal pathways in HEI-OC1 cells (Figs 3 and 4). The ROS scavenging ability of DEX was previously reported in murine cochleae intraperitoneally injected with DEX, in which biosynthesis of endogenous GSH was enhanced by the activation of γ-glutamylcysteine synthetase [38]. Moreover, DEX treatment increased the content of GSH and NADPH concomitant with the promotion of nuclear translocation of Nrf2, improving the antioxidant capacity in ataxia telangiectasia lymphoblastoid cells [39]. Considering the close association of ROS formation with the degeneration of cochlear cells, the therapeutic effect of DEX on TNF-α-induced ototoxicity could be related to redox homeostasis in auditory hair cells.

Treatment of auditory hair cells with ototoxic drugs, such as gentamicin and cisplatin, has shown increased expression of TNF-α mRNA [40, 41], and HC damages in cochlear explants [42, 43]. It has been also reported that treatment of TNF-α leads to disruption of the stereocilia bundle and damage to HCs in cochlear explants [30, 44]. In the present study, we found that TNF-α-induced HC loss in cochlear explants was proportional to its increased concentration (Fig 5). The otoprotective effect of DEX on ototoxic drug-induced HCs loss has been previously reported in explant culture studies. For example, HCs loss and caspase-dependent apoptosis induced by gentamicin were significantly attenuated in mouse cochlear explants via DEX pretreatment [45]. Also, pretreatment of DEX in cochlear explants significantly reduced cisplatin induced OHCs loss [46]. In animal model, intratympanic injection of DEX in mice significantly reduced the elevation of auditory brain stem response (ABR) threshold induced by cisplatin [47]. Consistent with these reports, we found that DEX pretreatment markedly reduced TNF-α-induced HCs loss and apoptosis in cochlear explants (Figs 6 and 7). Taken together, these results suggest that DEX pretreatment has a pivotal role in preventing TNF-α-induced HCs damage in cochlear explants.

## Conclusion

In conclusion, we have shown that DEX pretreatment protects TNF-α-induced ototoxicity in both auditory hair cells and cochlear explants. The otoprotective effect of DEX is achieved through attenuation of caspase-dependent apoptosis signaling pathway and ROS accumulation induced by TNF-α. Our findings broaden the understanding of the beneficial effects of DEX pretreatment on TNF-α-induced ototoxicity and also provide basis for designing therapeutic approaches to treat or prevent inflammatory-related hearing loss.

## Supporting information

S1 Raw images (PDF)
