# Supplementary material for: Dexamethasone treatment of murine auditory hair cells and cochlear explants attenuates tumor necrosis factor-α-initiated apoptotic damage
Source: PLoS One. 2023 Sep 21;18(9):e0291780. doi: 10.1371/journal.pone.0291780 (PMC10513268; doi:10.1371/journal.pone.0291780)
Supplement: S1 Raw images — (PDF) [file pone.0291780.s001.pdf]

Figure 2B. Original WB film images

Con: Control

DEX: Dexamethasone

M: Marker

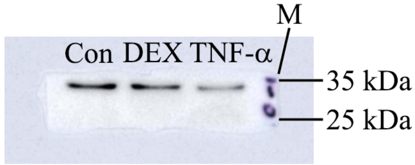

Figure 2B: Bcl-2

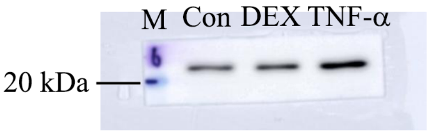

Figure 2B: Bax

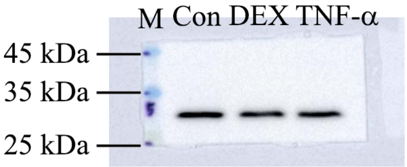

Figure 2B: Caspase 3

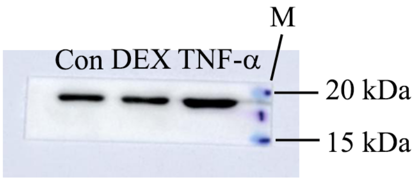

Figure 2B: Cleaved Caspase 3

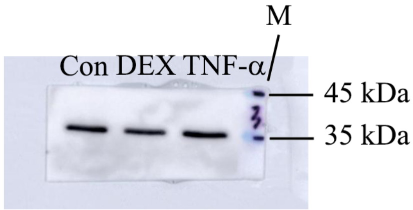

Figure 2B: Caspase 7

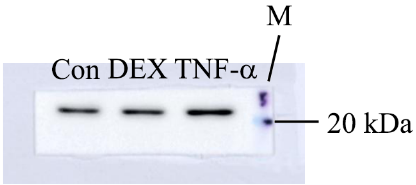

Figure 2B: Cleaved Caspase 7

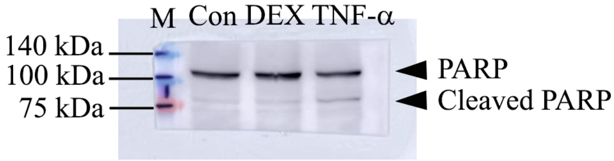

Figure 2B: PARP/Cleaved PARP

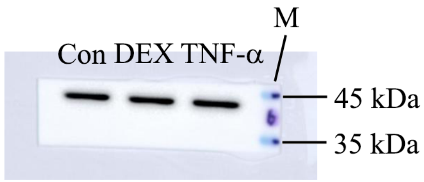

Figure 2B: β-actin

Figure 3C. Original WB film images

Con: Control

DEX: Dexamethasone

M: Marker

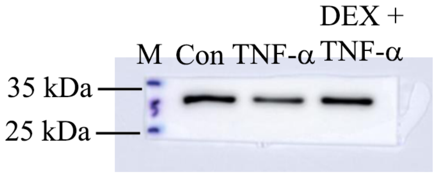

Figure 3C: Bcl-2

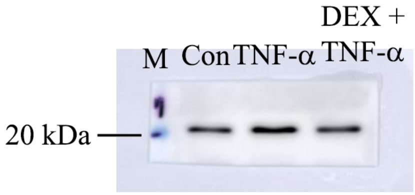

Figure 3C: Bax

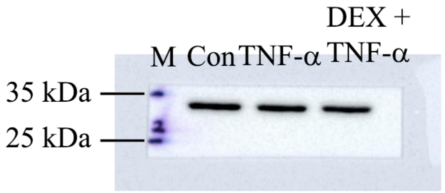

Figure 3C: Caspase 3

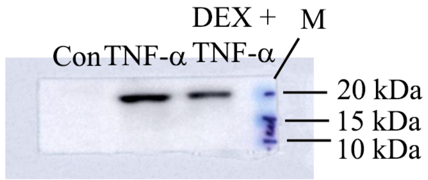

Figure 3C: Cleaved Caspase 3

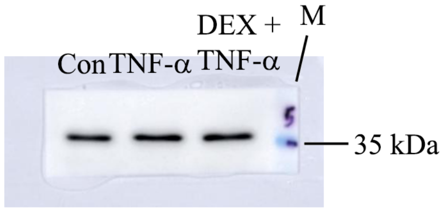

Figure 3C: Caspase 7

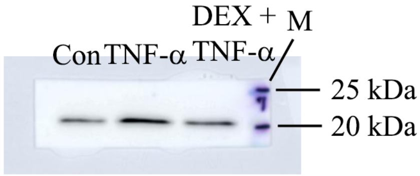

Figure 3C: Cleaved Caspase 7

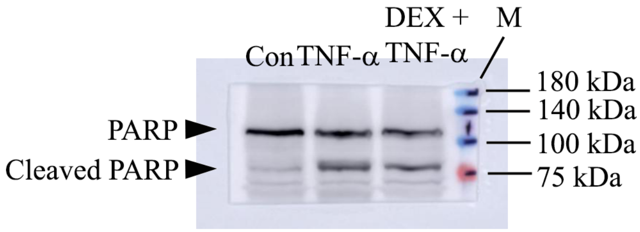

Figure 3C: PARP/Cleaved PARP

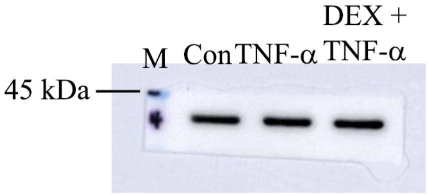

Figure 3C: β-actin
